# Editorial: Novel applications of epitope biology to improve outcomes in transplantation

**DOI:** 10.3389/fgene.2025.1572987

**Published:** 2025-02-26

**Authors:** James H. Lan, Robert Liwski, Alberto Cardoso Martins Lima, Sandra Tafulo

**Affiliations:** ^1^ Departments of Pathology and Laboratory Medicine, and Medicine, Division of Nephrology, University of British Columbia, Vancouver, Canada; ^2^ Department of Pathology, Dalhousie University, Halifax, Canada; ^3^ Histocompatibility Laboratory, Complexo Hospital de Clínicas, Federal University of Parana, Curitiba, Brazil; ^4^ Porto Blood and Transplantation Center, Portuguese Blood and Transplantation Institute, Porto, Portugal

**Keywords:** epitope analysis, transplantation, compatibility, matching, eplet

The launch of this Research Topic also marked the 70th anniversary of the first successful human kidney transplant performed at the Peter Bent Brigham Hospital (Harrison et al., 1956). In the decades that followed, transplantation evolved from an experimental procedure to the preferred live-saving therapy for those with organ failure, with >170,000 solid organ transplants performed worldwide in 2023 (Global Observatory on Donation and Transplantation). Despite this progress, alloimmune injury remains a major contributor to premature allograft loss (Gaston et al., 2010; Gourishankar et al., 2010; Einecke et al., 2009; Sellarés et al., 2012). While it is known that compatibility at genes encoding the human leukocyte antigens (HLA) is associated with superior transplant outcomes, the enormous polymorphisms of the HLA genes, now comprising over 40,000 alleles (HLA Nomenclature), make extensive matching impractical.

Advances in genome and proteome sciences have defined unique epitopes, regions of the HLA molecules defined by structure or charge, that are recognized by T-cells and antibodies and determine graft immunogenicity and antigenicity (Bjorkman et al., 1987; Zhang et al., 2005; Duquesnoy, 2014; Tambur and Claas, 2015). Clinical studies indicate that epitope mismatches are associated with graft rejection and inferior survival (Wiebe et al., 2013; Senev et al., 2020; Sapir-Pichhadze et al., 2015). While this measure does not improve matching, it may be leveraged to inform post-transplant monitoring and immunosuppression management. Other modeling studies suggest that the limited number of these epitopes, numbering only a few hundred, may permit new opportunities to optimize epitope matching during organ allocation to improve survival (Tran et al., 2021). These hypotheses offer new opportunities to improve outcomes but require rigorous evaluation, implementation of enabling technologies, and development of clear allocation policies. The focus of this Research Topic was thus to present novel approaches by which epitope biology could be used to improve the assessment of molecular compatibility and outcomes in transplantation.

Mattoo et al.’s comprehensive review is a timely assessment of the current landscape of molecular HLA and non-HLA matching in transplantation. Key approaches used to perform HLA compatibility analysis, including characterization of the risk of indirect T cell activation (Predicted Indirectly ReCognizable HLA Epitopes, PIRCHE (Geneugelijk and Spierings, 2020)), quantification of the amount of mismatched surface-exposed amino acids (HLA Matchmaker (Duquesnoy, 2006)), comparison of the physiochemical differences between HLA (Electrostatic Mismatch Score (Mallon et al., 2018)), and enumeration of the number of solvent-accessible amino acids (HLA Epitope MisMatch Algorithm, HLA-EMMA (Kramer et al., 2020)) were discussed and interpreted with data from relevant literature. The authors concluded with their views on the readiness (or lack of) of molecular compatibility assessment in specific clinical applications, and suggestions of areas that require further development and confirmation through clinical trials.

The original research study performed by Doxiadis et al. introduced the concept of graphical HLA eplet amino acid repertoire translation called epiArt. In the HLA community, the long-recognized patterns of cross-reactive groups have been defined by serologic reactivity (Rodey and Fuller, 1987). As our understanding and definition of the antigenic portion of the HLA molecule evolve, a new approach to visualize the relationship between eplets, small configurations of surface-exposed polymorphic amino acid residues, is required. To this end, the authors translated the amino acid sequences of antibody-confirmed eplets into an atlas of HLA class I and II antigens, followed by visualization of the pairwise allele distances by means of antigen-specific disparity graphs in differential amino acid space, showing intra-group heterogeneity of HLA class I and II alleles, as well as shared inter-group and inter-locus eplets and epitopes. This data revealed inconsistencies in the current HLA group nomenclature, indicating the need for an adjustment to how we contextualize similarities and differences between HLA alleles.

The majority of computational tools used to assess molecular compatibility require the input of high-resolution HLA genotypes. When this data is not available, the validity of using imputed HLA genotypes for molecular compatibility assessment remains uncertain (Engen et al., 2021). Matern et al.’s study evaluated the effect of imputing high-resolution genotypes on molecular mismatch scores under a variety of ancestry assumptions. The authors analyzed a simulated patient-donor dataset and confirmed using two real-world datasets. By comparing molecular matching scores from “ground-truth” high-resolution genotypes against imputed genotypes, the authors found that the use of multiple imputation and correct ancestry assumptions can greatly reduce error introduced during imputation. The authors concluded that for epitope analysis, imputation can be a valuable and low-risk strategy when accurate ancestry assumptions and the appropriate imputation strategy are applied.

Cellular therapies are increasingly investigated for different applications in transplantation. Yet, the potential risk of inciting immune responses against the donor allograft remains a relative concern. In the context of allogeneic mesenchymal stromal cell (MSC) therapy following kidney transplantation, Bezstarosti et al. investigated whether shared HLA epitopes and repeated amino acid mismatches between the kidney and MSC donor could trigger a donor-specific antibody (DSA) response. The study involved two cohorts (n = 20): one that selected MSC donors to avoid repeated HLA mismatches (Leiden) and another that did not (Liège). The key findings were that selective avoidance of repeated mismatches at the split HLA antigen level did not prevent repeated mismatches at the amino acid level. Despite this, repeated amino acid mismatches did not appear to increase the risk of DSA formation following allogeneic MSC therapy. Given the low rate of DSA detected in this study (3/20), confirmatory studies with larger cohorts would be warranted to define the immunogenicity of allogeneic MSCs.

In recent years, HLA-DR/DQ eplet mismatch has been associated with *de novo* DSA formation, rejection, and allograft loss, but Asian ethnicities have been under-represented in these study cohorts (Wiebe et al., 2019; Senev et al., 2020). Wong et al. performed a retrospective cohort analysis of 234 Southeast Asian kidney transplant recipients to evaluate HLA-DR/DQ eplet mismatch as a predictor of *de novo* DSA development. Single molecule eplet mismatch was quantified using HLA Matchmaker with prior eplet mismatch thresholds to categorize immune risk groups. They demonstrated that HLA-DR/DQ single molecule risk categories correlated significantly with *de novo* DSA-free survival. In addition, the authors identified slightly different thresholds in this predominantly cyclosporin cohort compared with previous studies that were tacrolimus-based, suggesting that the type of immunosuppressive therapy can potentially modulate the risk of eplet mismatches.

In summary, the articles of this Research Topic highlight the different approaches that epitope biology could be used to support compatibility and risk assessment in transplantation. As the methods of HLA epitope assessment continue to be refined and validated across broad ethnic populations and heterogenous immunosuppressive protocols, this Research Topic will play an integral part in the implementation of precision medicine and the next Frontier of immunosuppression minimization and tolerance in transplantation.
